# Reaping what you SOW: Guidelines and strategies for writing scopes of work for statistical consulting

**DOI:** 10.1002/sta4.496

**Published:** 2022-08-10

**Authors:** Ryan A. Peterson, Camille J. Hochheimer, Gary K. Grunwald, Rachel L. Johnson, Cheyret Wood, Mary D. Sammel

**Affiliations:** Department of Biostatistics and Informatics, Colorado School of Public Health, University of Colorado Anschutz Medical Campus, Aurora, Colorado 80045, USA

**Keywords:** collaboration, effective communication, planning, project management, scoping projects

## Abstract

Statistical consulting has the potential to be remarkably rewarding or frustratingly fruitless. Attentive and careful execution of a statistical scoping process will yield top-caliber research and collaborative partnerships. We provide guidelines and strategies for how to write effective statistical scopes of work (SOWs) and build this procedure into a statistical consulting workflow, using illustrative case studies along the way. Effective SOWs provide a solid foundation on which to build fruitful research partnerships, to avoid common pitfalls and to weather turbulent situations successfully.

## 1 | INTRODUCTION

Let us begin by defining a set of mutual expectations concerning your readership of this paper. Based on this paper’s title, you are likely expecting to learn the best available framework for initiating statistical consulting projects, with the hope that you will be able to learn about and implement some new strategies to boost your or your team’s productivity. We, this paper’s authors, have expectations of you as well; we expect you to understand terms such as statistical consulting and to be interested in how you can optimize your experience when participating in statistical collaborations. We also wish to note up front that due to the breadth of the field of statistics, our guidelines and strategies may not be a good fit for every consultant or consulting center. However, we hope that this work initiates a helpful conversation in statistical consulting circles, and we invite differing opinions to weigh in with other ideas and best practices for scoping a statistical analysis. Among the authors, we have a combined 70+ years of experience in statistical consulting in academics and have taught 33 MS-level courses in statistical consulting. Through this experience, together with guidance from 32 consulting statisticians and staff members at the Center for Innovative Design and Analysis (CIDA) in the University of Colorado, we have developed guidelines and strategies for effectively scoping statistical consulting projects. While perhaps not universally optimal, they are functional, and they are our (subjective) best practices based on our collective experiences. We hope they are helpful, facilitate good science and protect your sanity in future collaborations.

If you decide to skim or skip the rest of the paper at this point, we have already demonstrated the potential for our process. In most relationship-driven endeavors, including your readership of this paper and statistical consulting, an early establishment of a shared understanding of expectations can either build a strong foundation for success, or an efficient, frustration-free off-ramp for dissolution. This shared understanding of a consulting arrangement is the foundational intention of a scope of work (SOW) in the context of statistical collaboration. More formally, a SOW is a document drafted by a statistical consultant (or members of their organization) that defines a research problem in statistical terms and specifies a set of deliverables, conditions and expectations. Typically, after an initial meeting and potential follow-up communication, a SOW is sent to a primary investigator (PI), the domain-expert who is seeking statistical help, and the PI is requested to read and sign it before the project can formally begin. Establishing expectations and a plan for analysis is critical for success in collaborative biostatistics (Pomann et al., 2020); we proffer the statistical SOW as a means of doing this effectively and consistently. A well-thought-out SOW builds a strong foundation for success when success is feasible. Many poorly or non-scoped projects succeed (i.e., research questions are answered), but all too often they do so at the expense of overworking a statistician. Other consequences resulting from the lack of a proper SOW include mismatched expectations causing disrupted relationships, negative sentiment towards statisticians, poor estimates of type and amount of effort required, working on unhelpful problems, uncontrolled expansion of requests and generally unproductive research.

**FIGURE 1:**
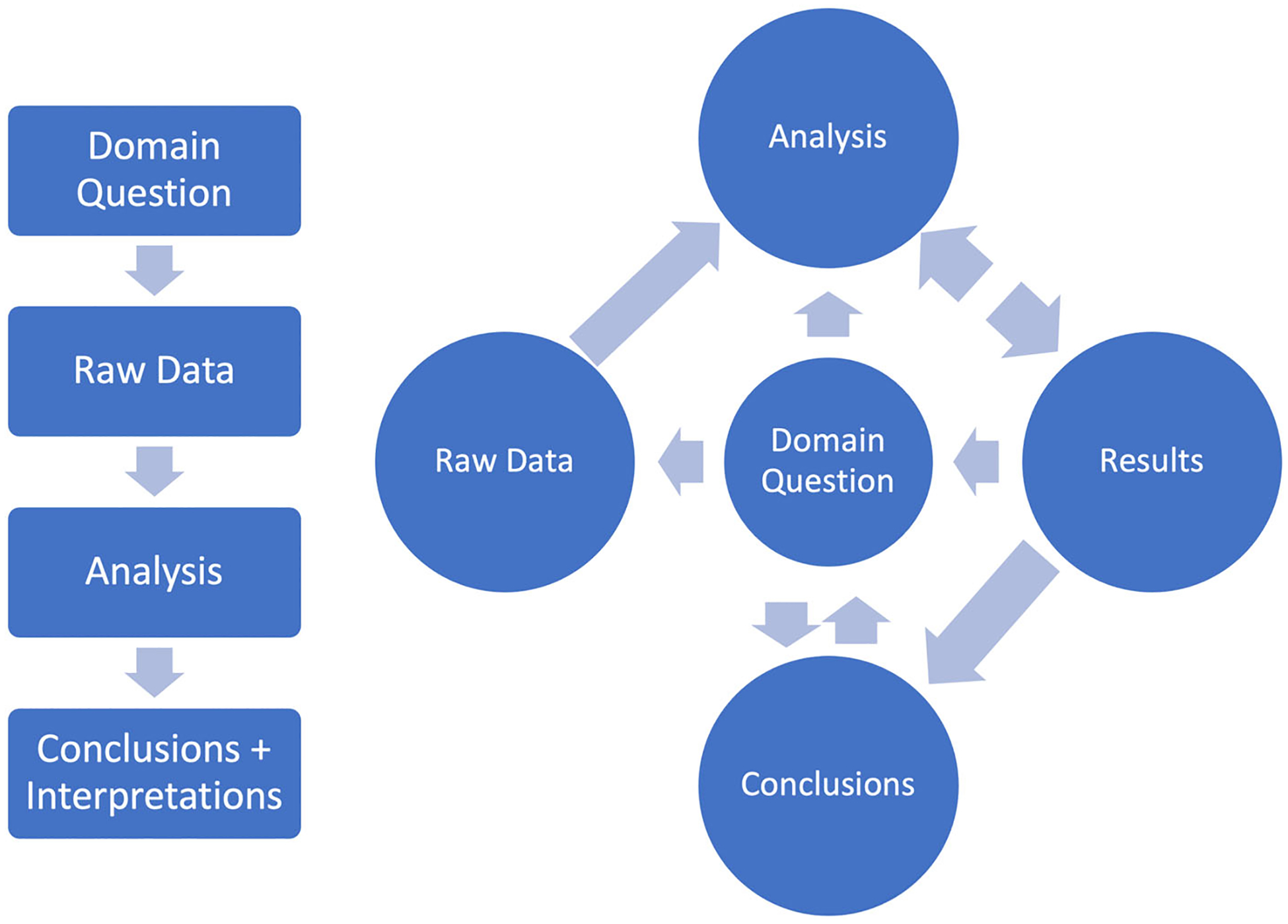
The utopian ideal for a statistical project workflow (left) versus reality (right)

The scoping process, while important, is neither simple nor effortless. Each project will typically have several complex constraints at play. What makes scoping statistical projects even more difficult is a popular fantasy of how research actually works. In an ideal world (the world in which most statisticians are trained), statistical work proceeds linearly according to the left of Figure 1: a primary investigator with a domain question produces data that we analyze to inform results and conclusions. The reality is messier (see the right side of Figure 1). Since research involves somewhat fungible hypotheses, methods and/or data, many statisticians have embraced reproducibility to ensure that the analysis can be updated efficiently and quickly, when necessary, for example due to slight changes in the data or methods. However, reproducibility is no panacea. Building a reproducible analysis workflow does help oil the research engine, but it takes time, effort and foresight to implement well. Even when done perfectly, additional requests and supplemental analyses can disrupt the best-laid plans. Worse, without strict statistical oversight, highly reproducible analysis workflows facilitate cherry-picking, p-hacking and the practice of making innumerable small, semi-innocuous analytical decisions to arrive at a “publishable” result (i.e., spending researcher degrees of freedom; Simmons et al., 2011).

The scoping process must respect and reflect the reality of an iterative research engine. While seasoned statistical consultants will understand how unrealistic it is to make hypothetical assumptions such as the left side of Figure 1, it can still be tempting to revert to such simplifying assumptions during the scoping process; a linear progression is much easier to plan for in terms of time, scope and budget and will come with a much lower price tag that will be easier for the PI to swallow. However, this assumption is a siren’s call, which, if heeded, leads to scope creep,^1^ mutual disappointment and burnout.

In the face of this conundrum, our center has developed a model process for project progression. Under this model, a project begins when the PI fills out an intake form. We then set up a scoping meeting and complete the SOW. Once the SOW is agreed to by the PI, the project begins in a kickoff meeting and proceeds with an initial preliminary report with a detailed analysis plan, a comprehensive report and an updated/revised comprehensive report, after which we provide manuscript support for 3 months. While the project progression model is wrong (like all models), in that most projects end up requiring slightly different deliverables and upkeep along the way, the model is especially *useful* when paired with an effective scope of work.

In this work, we outline eight guidelines for writing effective statistical SOWs. These guidelines are loosely ordered in a sequence of items to consider in any project scope. For each guideline, we describe several strategies we have found to be successful as well as case studies that illustrate its importance (i.e., lessons we have learned in the hard way). Then, we explain how we practically implement these guidelines, including our suggested scoping workflow, a list of questions to ask during this process and a SOW template. We conclude with a discussion that we hope will be a jumping-off point for a broader discussion within the statistics community.

## 2 | METHODS

### 2.1 | Guideline: Define study aims and decide whether the project is feasible early

*Strategy: Determine aims/objectives and PI’s needs at the project outset*. Ask the PI for their main objectives at the intake stage (as they see them) and early in the scoping meeting, so that objectives can be statistically refined. Consider potential secondary and sensitivity analyses when possible. Given this information, carefully determine whether there is sufficient time, budget and expertise available to meet the objectives. In some cases, expertise from other statisticians with relevant experience can be leveraged to estimate the scope of the PI’s research objectives more precisely. For larger organizations, this may require or be facilitated by leadership and/or project management staff. Also, if given the luxury, the SOW writer can consider whether the goals of the PI are in line with theirs or their center’s.

*Strategy: If not feasible, refer PI elsewhere (earlier is better, but avoid the sunk cost fallacy)*. Feasibility cannot always be decided right away; sometimes, this can depend on the quality and quantity of data, statistician or center-specific goals, or shifting resources and priorities. It is much better to end a doomed project before it begins, even if one has already invested time writing the SOW and looking at the data.

*Strategy: Describe the reasoning for outside referral to preserve the relationship*. Clearly communicate any perceived issues, as some feasibility concerns may be the result of a simple miscommunication. This can also guide the PI towards the next steps in their project.

*Strategy: Consider the scope/cost/time triangle*. Draw a triangle with vertices labelled scope, cost and time, and then explain to the PI that each vertex is constrained by the other two. Simple analyses with smaller scope (e.g., conference abstracts) can be accomplished more quickly or at lower cost, while comprehensive analyses (e.g., a manuscript) will take more time and funding to accomplish.

*Strategy: Do not scope projects estimated to take less than 5 h*. We have learned from internal data that writing a scope of work will take a minimum of 5 h of a trained analyst’s time. If a project could be completed in less time, the scoping process should be circumvented, for example, the PI should be referred to an hourly statistical consultant.

*Case study:* Derek, a statistician, has met with a PI, Sherrie, who hoped that someone could validate her analysis in a different statistical software. Sherrie explained that it should not take long, as she only had one code file (and a limited budget). Derek, understanding the project had narrow scope and funding, budgeted 1 month for the task. However, after the project kicked off, he discovered that the single code file contained hundreds of lines of uncommented code and several very large accompanying datasets. Although he completed the project, it took five frustration-filled months. Because of mismatched expectations along the way, neither Derek nor Sherrie felt eager to work with each other ever again. Lesson learned: Take the time to carefully review all available study materials while drafting the scope of work and do not assume a project will be simple just because the budget is limited. Rather, this project should have been scoped as a larger project, even if it was rendered non-feasible and the PI was referred elsewhere.

### 2.2 | Guideline: Delineate the practical and human expectations

Strategy: Collect all important deadlines and review expected timelines regarding the study objectives and the data of interest.

*Strategy: Agree upon a communication strategy and frequency for the course of the project*. We typically will provide one business day turnaround on communications, and we expect the same from the PI’s team unless otherwise specified.

*Strategy: Create list of personnel involved (study team and staff) and each person’s role*. It is especially important to get information for those who have worked with or collected the data, administrative staff, and, for junior researchers, contact information for their project mentor.

*Strategy: Inform PI of expectations for authorship and authorship discussions*. For example, *“*By providing the deliverables specified in this SOW, authorship will be conferred to analysts per the ICMJE guidelines (https://www.icmje.org/recommendations/).”

*Strategy: Discuss contingencies*. What happens if a deadline is missed? (Answer: The PI may reach out to leadership). What happens if data are not available, or if questions pertaining to the data cannot be answered quickly? (The statistician may request scope extension, and the project may be delayed or paused.) What happens if the research question does not show what the PI hopes it does? (The scope can be extended to run supplemental analyses, and this will require more funding.)

*Case study:* A medical student sought statistical support for his research project. At a scoping meeting, the student PI could not answer important details regarding his research goals, causing long delays in the scoping process. After the project had been completed, the analyst lost touch with the PI. However, she later discovered that her name had not been included in the author list for the conference poster despite her having produced several prominent presented results. Lessons learned: for junior PIs, request that their mentor(s) attend the scoping meeting and emphasize the importance of authorship for analysts. Consider sharing the PI-facing guidelines on expectations for statistical consulting from the Statistical Consulting Section of the American Statistical Association (https://community.amstat.org/cnsl/forclients/expect-content) during the original SOW meeting.

### 2.3 | Guideline: Define data expectations

*Strategy: Provide links to resources on spreadsheets* + *data formatting*. We make it a point to mention our own data formatting guidelines on our website (https://bit.ly/3lAv3mj), and we will also share a nice paper focused on common issues in spreadsheets (Broman & Woo, 2018).

*Strategy: “Glimpse” the data prior to finalizing scope of work*. If the data are already available prior to writing the SOW, it can sometimes be illuminating to examine them while drafting the scope or even during the SOW meeting. Diagnosing and fixing data quality issues takes time and effort, so if you find widespread data quality issues, this has important implications for the scope of work. A glimpse of the data is not analysis (wait to do more formal data examination and analyses until the scope of work agreement has been signed by the PI), but some cursory checks can be done quickly and are immensely helpful when scoping. Glimpsing the data might involve the following:

- Checking that all variables of interest are included in a standard, anticipated format.
- If data are spread in multiple tables, checking to make sure they have matching variables.
- Producing missing data summaries.
- Ensuring the sample size and number of variables of interest matches what PI says.

If issues are discovered in the data glimpsing process, clearly articulate data management needs and account for them generously in the SOW budget and timeline.

*Strategy: Inquire whether the PI has a full study protocol and/or a data dictionary available*. A study protocol, especially one that has been reviewed by a biostatistician (Ciolino et al., 2021), will give you more insight into the planned analysis, including specifics such as outcomes, surveys used and study design. Looking at the data dictionary will provide more insight into potential misunderstandings about the data structure. Connecting planned analyses from the study protocol with components of the data dictionary can illuminate issues (for instance, perhaps they did not collect something they had planned to analyze). We will typically ask this question before or during the SOW meeting.

Strategy: If data are not available, discuss the potential for the budget to increase and deadlines to shift should the data require more wrangling than expected.

*Case Study:* Stacy, a statistical analyst, was required to use a relational database for her analysis. In her SOW, she listed the typical time needed for creating an analysis dataset, and the project got picked up from there by another statistician. The project began on schedule but soon became very delayed; it turned out that the statistician required months to get his bearings with an unfamiliar database, validate the parameters of the data pull, investigate the data quality of the variables to be included in the analysis and work with the data vendor to clarify questions. Lesson learned: Include extra time for data management for relational databases, especially when first working with them.

### 2.4 | Guideline: Balance specificity and flexibility in a statistical analysis “sketch”

*Strategy: Do not be too specific; the statistical plan should be general enough to handle common contingencies/data issues*. We call the statistical analysis plan in our SOWs a “statistical analysis *sketch*,” recognizing that it should be non-prescriptive and serve as a loose guide for the analyst to paint their piece of statistical art. If details are too specific, the statistical analysis can be inflexible, and the productivity of the project becomes very sensitive to minor departures from expectations. Avoiding over-specificity will help analysts steer clear of spending their time solving unhelpful problems. Who pays for an analysis that answers the wrong question? Common contingencies to consider in the statistical analysis sketch include data management, variable screening based on missingness, data transformations, outlier detection and resolution, computation of variance inflation factors, model selection, regression diagnostics, prognostics and treatments.

*Strategy: Do not be too vague; the number of hypothesis tests (research questions) involved should be clear and written out explicitly*. While clarity is important, these should still allow for a flexible framework to analyze the data (e.g., no need to specify exact models). A trained statistician should be able to read the SOW and quickly envision how they could perform the analysis. Determine whether this research is exploratory or confirmatory; multiplicity adjustment may be warranted.

*Strategy: Make sure the SOW answers the question “at what point will this scope require expansion, in terms of calendar time or analyses requested?” In other words, “When would this start to become scope creep?”* To this end, aim for language to be flexible but detailed enough to recognize when scope creep *could* start occurring. If insufficient details are provided in the SOW, who will that affect? (Answer: either you or your soon-to-be-former colleague.)

*Strategy: Build template tables and figures*.

*Case study:* When Jason wrote a SOW for a project utilizing multiple linear regression, the data were not yet available. He asked several questions about the data types for the outcome and predictor variables and was told the outcome was continuous but they were not sure about the predictor variables. When he received the data at the project start, he discovered a high degree of multicollinearity among a very large set of predictor variables, as well as several large outlying/influential points that seem to be related to data quality concerns, rendering his SOW too narrowly defined. Lessons learned: When possible, confirm the terms of a SOW only after glimpsing the data. Otherwise, it is worth it to specify the number and types of variables of interest. These will help to clarify the research questions in the SOW. However, we recommend stopping short of assigning specific analyses in the SOW, even with data in hand.

### 2.5 | Guideline: Keep target audience in mind

The target audience includes the statistician(s) who will work on the project, the PI and administrative/research staff who will help manage the project and/or perform project budgeting.

*Strategy: Provide the necessary details and avoid excessive boiler-plate text*. A scope of work is a place for describing the background of the problem at hand, specifically outlining the research questions of interest and how they will be assessed/answered, describing the data structure and study design, specifying a sketch of the planned analysis methods, planning the tables/figures to be produced, defining the primary outcome and/or explanatory variables of interest as applicable and defining mutual expectations. These items establish a shared understanding of the project, its goals, its data and more. A scope of work is *not* a place for challenging the integrity of a study (unless this is requested), vaguely worded hypotheses (or “open” questions), preliminary results, discussion of a study’s limitations outside of how they relate to the statistical analysis, detailed descriptions about things that are not statistically relevant or copied/pasted sections from existing study materials.

*Strategy: Do not include jargon*. Jargon here may refer to statistical terms like “residuals,” “leverage,” “nuisance parameter” or “covariance matrix.” Too much statistical jargon may be helpful for the statistician but may distract, annoy or confuse the PI. Write to a PI who reads scientific papers but tends to skip over the methods section. Likewise, do not assume the SOW reader will know domain-specific jargon; write to a statistician with no domain expertise. Using common knowledge begets shared understanding, which is critical for success in collaborative projects (Vance et al., 2022).

*Strategy: Go through the full scope of work together in a kickoff meeting*. Statisticians and PIs are busy people and may not have time to fully digest the scope of work if it gets relegated to an email attachment. A kickoff meeting can help both the analysts (who are not necessarily the same ones who drafted the SOW), as well as the PI team retain information that is critical to the study’s success.

*Case study:* During a SOW meeting, Thando was working with a PI’s study team to clarify their project’s research goals. Despite asking several pointed questions about hypotheses and outcomes, she was not getting enough specific information to isolate clear research questions. She gently reminded the domain experts that statisticians have expertise in study design and suggested they start with a smaller SOW focused on study design to further develop their research questions and hypotheses. Lesson learned: If a statistician cannot reasonably outline analyses from the original hypotheses posed, consider starting first with a separate SOW for study design, building time into the SOW for solidifying clear hypotheses or referring PIs to hourly consultations where their research questions can be sufficiently refined.

### 2.6 | Guideline: Stay organized and use the time efficiently

*Strategy: Follow POWER guidance for structuring the scope of work meeting*. The POWER framework stands for Prepare, Open, Work, End and Reflect (Zahn, 2019). This framework helps facilitate well-structured meetings that build relationships and that beget critical information about the study.

*Strategy: Use the ASCCR Frame, and make sure to ask great questions*. The ASCCR framework stands for Attitude, Structure, Content, Communication and Relationship (Vance & Smith, 2019); it is exceptionally useful for training statistical analysts how to run scope of work meetings. Importantly, related to “communication,” follow-up questions should be strategically worded to simultaneously create shared understanding and build trust.

*Strategy: Take notes and ensure all vital questions have been answered by the end of the meeting; otherwise, schedule a follow-up scoping meeting*. Vital questions are listed in Table 1 in the next section and are based on the conglomeration of strategies in this paper.

*Strategy: Use a template*. CIDA uses a template that we have made available as supporting information. We are continually updating and changing this document as we learn new lessons. Remember, however, that boiler-plate text gets skimmed or missed. Do not assume PIs will read the full SOW; rather, walk them through it in detail at the initial project kick-off meeting.

*Case study:* Late on a Friday afternoon, Gerald, a staff statistician, met with a new PI for a scoping meeting. While he thought the meeting went well and he used a template to ensure all vital information was communicated, he was not at his usual workspace and could not take good notes. He then got sick over the weekend and was finally able to return to work a week later, at which time he could not recall much of the relevant information. As a result, the scope was further delayed, and the PI had to spend redundant time re-answering several questions. Lesson learned: Take organized, contemporary notes during and after the scoping meeting, using the exercise as a means of asking important follow-up questions. Send a follow-up email to the PI reviewing the main takeaways from the meeting to make sure you are both on the same page.

### 2.7 | Guideline: Empower PIs

*Strategy: Provide clear directions for obtaining administrative support and getting in touch with leadership or supervisors*. To build successful collaborations and healthy working relationships, neither party should be powerless to address roadblocks. PIs should understand that they are valuable members of the analysis process and will be respected. Knowing who to contact for questions related to project delays, billing issues or other problems is necessary to this end.

*Strategy: Build mechanism for* ***actively soliciting*** *both positive and negative feedback from clients*. For example, send emails with a link to a survey after the intake form, scoping process and project conclusion. PIs with strong negative interactions *will send* feedback to leadership anyway, so active solicitation encourages positive feedback, while illuminating potential bottlenecks/pain-points in the analytic process from less vocal PIs. In a similar context, BERD authors have stressed the importance of collecting metrics pertaining to PI satisfaction and suggest collecting feedback for timeliness, professionalism, collegiality, efficiency and knowledge/skills (Rubio, et al., 2011).

*Case study:* Isabella had been excited to enlist the help of a new statistical consulting organization (NSCO) in answering her research questions. She was impressed with the NSCO analyst she met with during the scoping process (Beck) and eagerly agreed to the SOW. However, after the project began, the statistician assigned to her project (Gerald) did not have a skillset well suited to her project, causing him to be unresponsive and behind schedule. She re-read her SOW document and found the contact information for NSCO leadership, so she reached out to them describing her situation and pointing to the SOW. The NSCO leadership team served as a mediator between Isabella and Gerald decided that the pairing was not a good fit and re-assigned Isabella’s project to Beck. Lesson learned: certain pairings of PIs and statisticians can be unpredictably vexatious. Providing PIs with guidance for communicating with center leadership can prevent breakdowns and negative sentiment, while sometimes even preserving fruitful relationships.

### 2.8 | Guideline: Empower statisticians

*Strategy: Train statisticians how to spot and deal with scope creep*. While we tend to eschew the term “scope creep” in formal training, the concept can be framed in terms of project prioritization with time/effort constraints. It is helpful to explicitly define an outcome hierarchy, that is, a very small number of primary outcomes, and perhaps a larger number of secondary or tertiary outcomes. Additionally, the statistician needs to feel comfortable being clear with the PI about how much time things take, for instance, if (when) data management takes longer than expected.

*Strategy: Provide topcover*. Topcover is the expectation that leadership will stand behind statisticians, encouraging them to object (politely) to requests beyond the agreed-upon SOW.

*Strategy: Build low-friction expansion mechanisms into SOW*. Such mechanisms will be especially useful for scope-creep-susceptible projects (e.g., those with less clear data needs or when the PI indicates a propensity to add supplemental analyses). For example, include the statement “additional requested analyses may necessitate a scope extension, which will be attached as appendix to this SOW and re-signed by both parties.”

*Strategy: Set authorship expectations right away in anticipation of a future line drawn in the sand*. It should be emphasized in the SOW that by providing the agreed-upon deliverables, authorship will be conferred to analysts per the ICMJE guidelines (see Section 2.2). Then, if the statistician must draw a line in the sand, they can still expect authorship.

*Case study*: Kate, the consulting center manager, noticed that a project with a study design SOW was still active 1 month past the intended end date. After checking in with Ben, the project statistician, she learned that the PI had requested preliminary analyses, some of which Ben had performed. Wanting to protect Ben’s time, Kate helped him craft an email politely declining further requests and redirecting the PI to the SOW team to scope out the additional analysis needs for the project. **Lesson learned:** Empower statisticians to say no when they are being asked to do extra work not covered under the SOW, *no matter how small the request*. Make sure that smaller SOWs are small projects, rather than a means to get poor Ben to do free consulting.

## 3 | RESULTS

The guidelines in the preceding section and their strategies were illustrated using mostly negative case studies, but thankfully, after learning these lessons, our practice has improved, and we now are able to write approximately 60 scopes per year. In this section, we present the various ways we have enshrined these guidelines in our practice.

**FIGURE 2:**
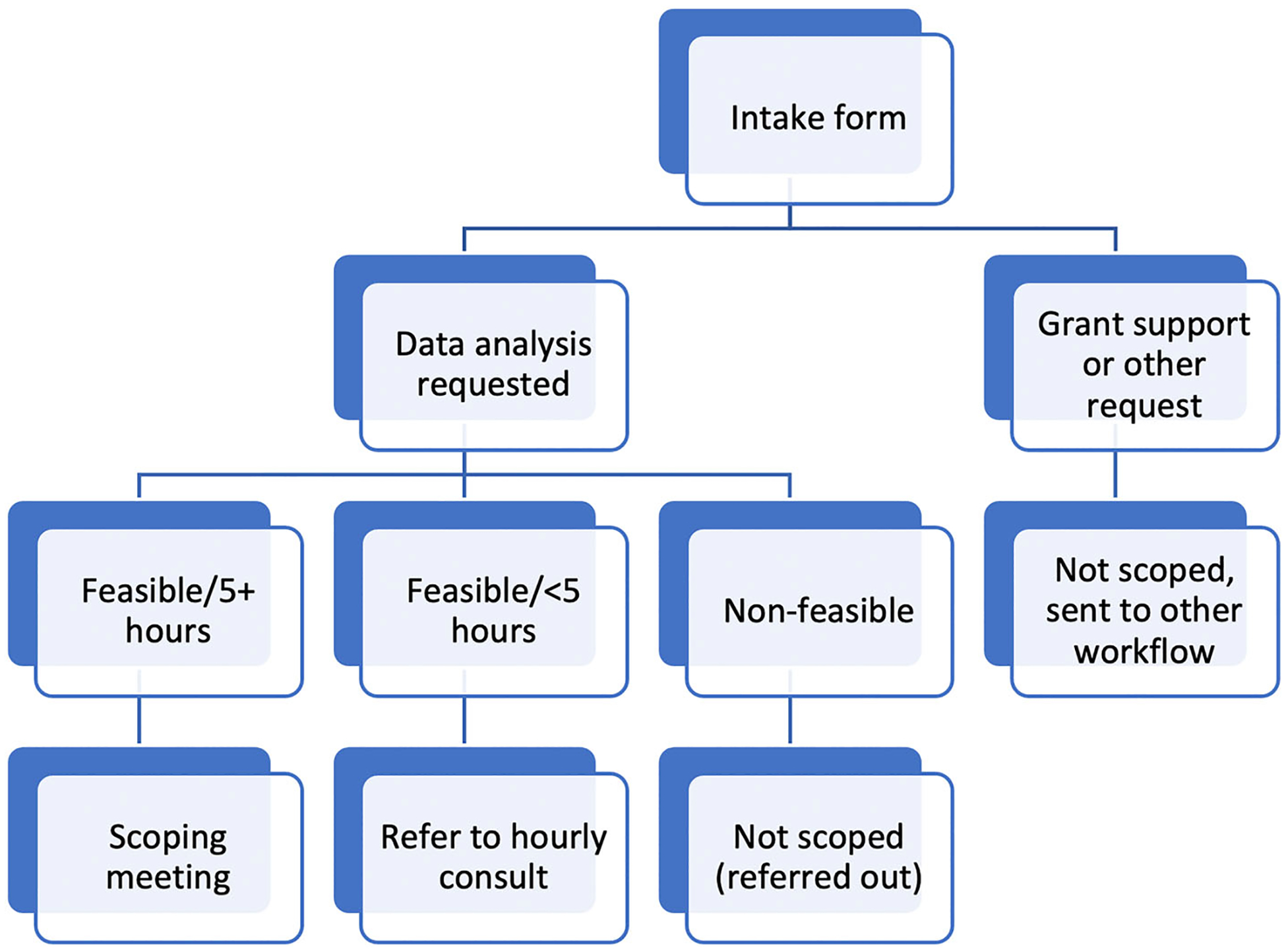
Our center’s process for determining which projects should undergo a scoping process

First, we now only scope certain types of projects. The intake form in Table 1 can be exceedingly helpful in this regard as it requires minimal oversight; projects where data analysis is not requested are rarely going to undergo the scoping process in the first place. The intake form helps us also triage other types of requests such as protocol reviews and grant proposal requests, which have separate mechanisms outside of the scope of this paper. When data analyses are requested, projects deemed feasible and sufficiently large will be scoped. We use this second screening procedure because, as we have noted, properly scoping a project can involve a non-negligible amount of time. At a minimum, we estimate this process will involve 5 h of work. We reject projects we do not believe are feasible, and we refer projects estimated to take less than 5 h to hourly consults. This process is illustrated in Figure 2.

Second, we request answers to each question listed in Table 1. While some items can and should be assessed in the intake form, we have found some questions are best asked verbally in the scoping meeting itself, and it helps to verify some of the information provided to ensure it matches the PI’s current plan. Each question in this list is there because of a guideline in Section 2. Importantly, many questions assess feasibility (early), gather expectations regarding the data, outline study personnel involved and define practical (human) expectations. Finally, we have put together a template SOW document which the analyst charged with scoping a project will fill out as a part of the scoping meeting. This template consists of a background, description of the data, research questions or hypotheses, a statistical analysis plan, an anticipated budget and timeline and, finally, a list of mutual expectations. We have included this template as supporting information.

## 4 | DISCUSSION

Statistical consulting can be an immensely fulfilling component of research, and it is ever more important as data sets get bigger, richer and more complicated to work with. Our experience with statistical consulting has evolved into a practice of writing effective SOWs based on the guidelines and practices we have outlined in this paper. While this process is not a cure-all solution to problems that can sometimes arise for statistical projects or collaborative relationships, we firmly believe that a good SOW will facilitate shared understanding and transparency of expectations, which in turn will provide a solid foundation for better research and richer professional relationships. We understand that due to the subjective and heterogeneous nature of statistical consulting, not all processes can or should look like the ones we have adopted in CIDA. However, while these guidelines and strategies are somewhat tailored to statistical consulting in an academic research environment, we hope they will be helpful in other settings and even spur a larger conversation about best practices for planning statistical projects in a consulting setting.

## Supplementary Material

SOW Suppliment

## Figures and Tables

**TABLE 1:** Vital questions to ask during the scoping process for statistical consulting projects

Intake form phase What can we help you with? (Statistical support for data analysis, 1-h consultation, grant development/study design, other) Gather background on requester (series of text boxes) Are your data currently available? Where and how have your data been collected? (REDCap/Paper charts/Excel/EMR/other) Do you have funding available for the project? If so, what type? Project information (text boxes) What are your upcoming deadlines? What is your anticipated budget for the statistical analysis? Scoping phase (for projects deemed feasible and 5+ h) Verbally describe your project and anticipated needs. Describe your [planned] data set(s). How many rows? How many columns with pertinent variables? Special cases: (for large data sets only) do you have computational resources for the analyst? If not, where and in what form are the data currently stored? Discuss a plan to deliver the data. (If >1 data set) How are data sets connected/related to each other? For pertinent variables identified in (2), what type are they (numerical or categorical), and are they outcomes or explanatory variables? If a variable is a survey score, is this score validated? Are the score(s) and variables calculated already? How much data management is required? (If determining information in (2) and/or (3) was difficult, the answer is almost certainly a lot.) Have you worked with these data before? Are there any restrictions on handling of these data (e.g., must they remain on a server?) Is there a point person who can answer questions regarding these data in a timely fashion? Are observations independent? If not, describe dependence (e.g., clustered and repeated measures) What are you hoping to show in your project? (Describe primary outcome(s) and hypotheses). Do you have a data dictionary and/or study protocol available? Will our default set^a^ of deliverables be sufficient for your needs? What is a good timeline for each deliverable? What end product are you seeking? (Manuscript/abstract/other). If we do not find the result(s) you are hoping to see in our analysis [Q9], or if the results run counter to your expectations, what would be your preferred course of action? (Submit manuscript anyway, investigate different analyses/outcomes,^b^ present as abstract but no publication, discard project, other) Who else is involved on the paper/project, and what are their roles? Important statements to mention prior to the meeting conclusion: We expect agreed-upon authorship roles and reasonable communication turnaround, and for the PI to read and agree to SOW. If the agreed-upon expectations are met, you can expect us to accomplish items listed in the SOW within the agreed upon timeline.

a We would describe our typical project structure is to produce an initial preliminary report and detailed analysis plan, a comprehensive report and one updated report. We will provide manuscript support for 3 months following the completion of the updated report.

b Note that additional analyses may require a scope extension.

## Data Availability

Data sharing is not applicable to this article as no new data were created or analyzed in this study.

## Footnotes

1 Scope creep refers to the tendency for statistical projects to advance gradually beyond usual or desirable limits.
