# Supplementary material for: Reaping what you SOW: Guidelines and strategies for writing scopes of work for statistical consulting
Source: Stat. Author manuscript; Available in PMC 2025 Jul 28. (PMC12302940; doi:10.1002/sta4.496)
Supplement: SOW Suppliment [file NIHMS2020364-supplement-SOW_Suppliment.docx]

# Scope of Work (SOW) Agreement

The goal of this document is to guide an analyst through a SOW meeting with an investigator in order to gather the information needed for a comprehensive SOW. Each section includes questions aimed at collecting information relevant to that section as well as reminders about expectations and timelines. Not every question is relevant to every project, nor is this an exhaustive list. Be sure to review the information provided in the intake form to avoid asking redundant questions or confirm them instead of asking them. This template, especially the Project Deliverables and Investigator Responsibilities sections, were created specifically for our center (the Center for Innovative Design and Analysis; CIDA) and will likely need to be edited to comply with other organizations’ services and policies.

## General Information

| **Investigator** | First Last first.last@university.edu | **Date** | June 23, 2022 |
| --- | --- | --- | --- |
| **Project Number** |  |  |  |
| **Project Title** | Title | | |

## Project Description

### Background

- Tell me about your project and analysis needs. What can we help you with?
- Do you have funding available for the project? If so, what type? What is your expected budget for statistical analysis?
- Who else is involved in this paper/project and what are their roles?

### Study Design

- What study design was used (e.g., RCT, prospective, cohort, case-control)?
- Do you have a protocol available? If not, please supply further details about your study design.

### Description of the Data

- Are your data currently available?
- Where and how have your data been collected? (REDCap (Research Electronic Data Capture), paper charts, Excel, EHR (Electronic Health Records), claims, other)
- Describe your data (screen share when possible)
  - How many tables/data sets?
    - If there is more than one dataset, how are they connected/related to each other?
  - How many rows?
  - How many columns/variables?
  - Which variables are relevant for analysis? What type of data are they (numerical, categorical)?
  - Which variables measure your outcome(s), and which measure other explanatory variables?
  - Are there scores included in your data? Are these scores validated? Have they already been calculated, or do they need to be calculated? If they need to be calculated, do you have the scoring?
  - Are observations independent? If not, can you describe the data dependence (e.g., clustered, repeated measures)?
- Where and in what format are the data currently stored? Discuss a plan to deliver the data.
- Have you worked with these data before?
- Are there any restrictions on handling these data? Do these data need to remain on a specific server? If applicable, do you have computational resources for the analyst?
- Is there a point person for answering questions about the data promptly?
- Is there a data dictionary available?
- What are the data management needs for this project?
  - Number of files
  - Have any data quality checks been done?
  - Note – if we receive the data and data management needs are more in depth than expected, this will result in an increase in the scope/cost of the project.

### Anticipated Sample Size / Study Population

- If you’ve already collected your data, how many subjects/observations do you have? Observations per subject?
- If you haven’t already collected your data, how many subjects/observations do you expect to have?

### Hypothesis

- What are the aims of your research?
  - Note: If the aims are not well-defined, consider whether a study-design-focused scope of work or hourly consulting is more appropriate.
- What are you hoping to show in your results?
- What is the intended end product of your project (e.g., manuscript, abstract, poster)?
- If we do not find the effect(s) you are hoping to see, or if the results run counter to your current expectations, what would be your preferred course of action? (Submit anyway, abstract instead, investigate different analyses/outcomes understanding that this will involve a new SOW, discard the project, other)

### Further Project Details

- Will the project deliverables below be sufficient for your needs?
- What is a good timeline for these deliverables?
- Are there any deadlines that we should be aware of (even if we can’t meet them)? Reminder that communication lags/delays in data delivery may affect this timeline.
- Our expectations include authorship, reasonable communication times, and agreement to the parameters (including limitations) of this SOW.

## Project Deliverables

1. **Exploratory Analysis Report**
   Following the project kickoff meeting, the analyst(s) will establish a draft analysis plan, create preliminary analysis dataset, create 1-2 descriptive statistics tables (Table 1 type tables in AMA format), produce graphics where applicable (up to 4), conduct exploratory analyses (e.g., run univariate models), and create 1-2 tables describing results. The Exploratory Analysis Report includes a brief section on the data (e.g., defining any variables for creation, subjects excluded and why, and other data manipulations needed for the analysis dataset), a draft analysis plan, and results of this preliminary analyses including the Tables and Figures. We require an in-person or zoom meeting to discuss these results and finish the analysis plan.
2. **Comprehensive Analysis Report**
   A final analysis dataset will be updated per any changes discussed during review of the exploratory report, and descriptive tables will be updated accordingly. Comprehensive analysis will be conducted according to the finalized analysis plan, and the analyst(s) will populate the 1-2 results tables, create up to 4 near publication quality graphs and update the analysis report. The Comprehensive Analysis Report includes an introduction, brief section on data, statistical methods section, and results section including Tables and Figures. We require an in-person or zoom meeting to discuss these results.
3. **Comprehensive Analysis Report Revisions**
   The analyst(s) will supply one update to the Comprehensive Analysis Report as needed to address points of clarification and small additional questions; these updates will be discussed during the review of the Comprehensive Analysis Report. Please note that more hypothesis testing beyond the agreed upon scope of work may result in added charges.
4. **Manuscript Editing**

The analyst(s) will provide edits to manuscript draft, to including a review of the manuscript for overall content and clarity, appropriately editing the statistical methods and results sections from the Comprehensive Analysis Report, and up to two updates of the figures / tables per journal requirements for the submission. Please note that **the manuscript draft should be provided to the biostatistician within 90 days after receipt of the Comprehensive Analysis Report revisions unless otherwise agreed upon**. Manuscripts submitted for editing after 90 days of receipt of the Comprehensive Analysis Report revisions may be subject to added charges. The analyst(s) will provide reasonable edits and draft responses to address reviewer questions and comments; however, additional work, including additional analyses requested by reviewers, may result in additional charges.

## Investigator Responsibilities

### Provide cleaned data: Unless data management is stipulated in the deliverables above, the data are assumed to be cleaned and ready for analysis. Please see our data format guidelines ([Data Format Guidelines](http://www.ucdenver.edu/academics/colleges/PublicHealth/research/centers/CBC/resources/Statistics-Tutorials/Pages/Dataset-Format-Guidelines.aspx)) for assistance if desired. Datasets that do not comply with the formatting guidelines will either be returned to the investigator or subject to additional fees for data management.

### Add analysts name to the IRB prior to sending or granting access to data.

1. **Remove any protected health information (PHI) or similar identifiers** from data that are not required for analysis, creating unique identifiers instead (e.g., MRN numbers).
2. **Be responsive** in email (1 working day) and be available for meetings during standard working hours throughout the project period.
3. **Include the analyst(s) as co-authors** where the analytic component of the project meets authorship guidelines. Our group abides by the [International Committee of Medical Journal Editors (ICMJE) guidelines concerning authorship](http://www.icmje.org/).
4. **Manuscript submission:** A draft of the manuscript is expected to be shared with the analyst team within 90 days of receipt of the comprehensive analysis report, and the manuscript to be submitted within 120 days. The investigator is responsible for taking the lead on writing all manuscript sections and making cosmetic/formatting changes to tables.

**Timeline / Deadlines**

The anticipated project start date is **08/1/2022** and revisions to the Comprehensive Analysis Report are expected to be complete by **9/1/2022**. Approximately one month prior to the project start date, the assigned analysts will reach out to the investigator to confirm the project timeframe and the project start date may be altered if necessary.

A kickoff meeting is to occur no later than **8/5/2022.** At least 1 week (7 business days) prior to the kickoff meeting, the Investigator will provide the analyst(s) with the dataset and other relevant study documentation. During the kickoff meeting, the project team will discuss and review project materials, outline and mockup the desired tables and figures (1-2 descriptive tables, 1-2 analysis results tables and up to 4 graphs), outline the data analysis objectives. During the kickoff meeting, the team will establish a meeting schedule for the duration of the project.

**Estimated Cost**

This represents our best estimate of the effort needed to complete the project. Should the scope of work change substantially, the analyst(s) will discuss changes with the investigator and issue a new or amended project agreement.

|  | Effort | | |
| --- | --- | --- | --- |
| Months | PhD Faculty | Master’s Faculty | Student |
| 1 | XX% | XX% | XX% |
|  |  |  |  |
|  |  | **Total Costs** |  |

Please Remember: The manuscript draft must be provided to the biostatistician within 90 days after receipt of the Comprehensive Analysis Report revisions, otherwise an amendment with additional charges may be required.

**Billing Details**

We will bill in the following manner: (insert billing practices here).

For questions regarding project billing, please contact XXXX@university.edu.

### Right to cancel or close out a project: SOWs not approved within 30 days will be closed. Projects which remain inactive for over 60 days will be closed unless prior arrangements have been made, and a final bill will be sent.

**Approval of Agreement**

By approving this Scope of Work Agreement, you are acknowledging that you have read and agree to the project costs and milestones, timelines, project details, and terms and conditions outlined in this document.

**To approve this Scope of Work Agreement, click the link in the email you received.**

You will need to accept the terms and conditions and update your billing information.
